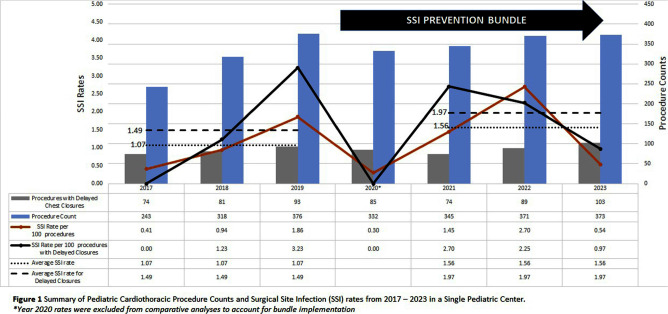# Sustainability of Surgical Site Infection (SSI) Prevention Bundle for Pediatric Cardiothoracic Surgery Patients

**DOI:** 10.1017/ash.2024.320

**Published:** 2024-09-16

**Authors:** Melissa Campbell, Jennifer Turi, Cheyenne English, Sharah Collier, Vani Sistla, Jessica Seidelman, Becky Smith, Sarah Lewis, Ibukunoluwa Kalu

**Affiliations:** Duke University Hospital; Duke University Health System; Duke University; Duke University Medical Center

## Abstract

**Background:** Frequent use of delayed sternal closure and prolonged stays in critical care units contribute to surgical site infections among pediatric patients undergoing cardiothoracic (CT) procedures. Bundled interventions to prevent or reduce surgical site infections (SSIs) have shown prior success, but limited data exist on sustainability of these efforts especially during the Coronavirus Disease 2019 (COVID-19) pandemic. Here, we re-examine the SSI rates for pediatric CT procedures after the onset of the pandemic. **Methods:** In a single academic center providing regional quaternary care, we created a multidisciplinary CT-surgery SSI Prevention workgroup in response to rising CT SSI rates. Bundle elements focused on daily chlorhexidine bathing, environmental cleaning, monthly room changes, linen management, antimicrobial prophylaxis, and sterile techniques for beside and operating room procedures. CDC surveillance definitions were used to identify superficial, deep or organ space SSIs. To assess the bundle’s sustainability, we compared SSI rates during years impacted by the COVID-19 pandemic (2021–2023, period 2) to pre-pandemic rates (2017–2019, period 1). Data from 2020 were excluded to account for bundle implementation, pandemic restrictions, and a minor decrease in surgical volumes. Rates were calculated as surgical site infection cases per 100 procedures. Mean rates across both periods were compared using paired t-tests (Stata/SE version 14.2). **Results:** Excluding the year 2020, the average SSI rate per 100 CT procedures increased from 1.07 in period 1 to 1.56 in period 2(p=0.55). Concurrently, the average SSI rate per 100 CT procedures with delayed closures increased from 1.49 in period 1 to 1.97 in period 2(p=0.67). Figure 1 shows SSI rates and procedure counts for 2017–2023. Coagulase negative Staphylococci most frequently caused SSIs in period 1 while methicillin-susceptible Staphylococcus aureus (MSSA) was most frequently identified in period 2. During period 2, the estimated compliance with SSI prevention bundle remained stable and reached 95% for pre-operative chlorhexidine baths and use of appropriate antimicrobial prophylaxis. Monthly room changes with dedicated environmental cleaning reached 100% compliance. **Conclusion:** Despite staffing shortages and resource limitations (e.g., discontinuation of contact isolation for MRSA colonization) during the COVID-19 pandemic, SSI rates for pediatric CT surgeries showed a slight, but non-statistically significant, increase in post-pandemic years as compared to pre-pandemic years. implementation of bundled interventions and improved surveillance methods may have sustainably impacted these SSI rates. Reinforcing bundle adherence as well as identifying additional prevention interventions to incorporate in pre-, intra-, and post-operative periods may improve patient outcomes.

**Figure:**